# O03 Superbug showdown: data versus microbes

**DOI:** 10.1093/jacamr/dlag102.003

**Published:** 2026-06-26

**Authors:** Helen Callaby, Rebecca Stout, Laura Ciaccio, Flana Baretto, Sally Forrest, Sarah Gallichan, Allan Zuza, Christian Cole, Jenny Johnston, Rachel Edwards

**Affiliations:** Population Health, Faculty of Health, University of Dundee, Dundee, UK; University of St Andrews, St Andrews, UK; Population Health, Faculty of Health, University of Dundee, Dundee, UK; Population Health, Faculty of Health, University of Dundee, Dundee, UK; University of St Andrews, St Andrews, UK; University of St Andrews, St Andrews, UK; University of St Andrews, St Andrews, UK; Health Informatics Centre, Faculty of Health, University of Dundee, Dundee, UK; Health Informatics Centre, Faculty of Health, University of Dundee, Dundee, UK; Research Data Scotland (RDS), Edinburgh, UK

## Abstract

**Background:**

Antimicrobial resistance (AMR) remains a significant public health threat, yet public understanding, especially among young learners, remains limited. Public engagement is included in the UK AMR National Action Plan (NAP).

**Objectives:**

To develop a public engagement event to educate children and their caregivers about how healthcare data is used in infection research, using the example of AMR. Key learning objectives were (i) how researchers apply for, access, and use de-identified public sector data, and how this can be harnessed to understand AMR; and (ii) how bacteria acquire resistance to antibiotics and how to prevent this.

**Methods:**

An interdisciplinary team of infection researchers from the Universities of Dundee and St Andrews, together with secure data professionals from Research Data Scotland (RDS) and Health Informatics Centre (HIC) co-designed and delivered three interactive activities. Activities were based on previously established activities designed by RDS (LEGO, card match) and Liverpool School of Tropical Medicine (Bacterial Supervillain at Bluedot Festival). The intended audience were children and their care givers at the Dundee Science Festival. Feedback from children was sought through a ‘sticker feedback sheet’ where children were asked at the beginning how much they knew about ‘superbugs’ before and after the activities.

**Results:**

*Data linkage using LEGO^®^ (age 7+):* We used LEGO to explain the role of a researcher accessing and linking data. Locked treasure chests represented the ‘Trusted Research Environment’ (TRE) and LEGO represented datasets. Children used the LEGO to represent the data being linked. Before playing with the LEGO they had to agree to be a safe researcher, to only conduct the research within the TRE, and design their research question which was built around choosing an infection, antibiotic and health condition to study. *Build a bacterial supervillain (age 4–10):* Children learnt about how resistance mechanisms are acquired through building their own bacterial supervillain. Using craft materials they chose the site of the body the organism affected, the shape of the bacteria (cocci or bacilli), mechanisms of resistance which gave them superpowers (such as a pipe cleaner for a flagella), and how the bacteria had spread (such as not washing hands). *Card match (age 12+):* A card match activity was designed consisting of 16 cards to represent data linkage. There were four patient scenarios, each with a unique patient identifier, age, symptom, infection and antibiotic (or no antibiotic). Participants were asked to match cards according to the unique identifiers. The game also helped to highlight scenarios where antibiotics are not required, such as for viral illnesses and *Escherichia coli* 0157. A total of 774 people attended the Science Festival. Feedback from children showed an improvement in knowledge after the activities. Most children arrived knowing very little about superbugs but left feeling informed, with feedback suggesting they now know ‘a little bit’ or even ‘a lot’ after taking part.

**Conclusions:**

This event showcased strong collaboration across universities and between data science teams. Activities were well received with positive feedback. This approach and materials codesigned could, and should, be replicated nationwide.

